# P-735. Healthcare Experiences of Sexual Minority Men with Severe Mpox During the New York City Outbreak: Lessons from In-Depth Interviews

**DOI:** 10.1093/ofid/ofaf695.946

**Published:** 2026-01-11

**Authors:** Sammy Plezia, Victoria Lanza, Sahnah Lim, Dustin Duncan, Jason Felder, Ofole Mgbako

**Affiliations:** University of Oregon, Eugene, Oregon; NYU Langone Health, New York, New York; NYU Grossman School of Medicine, New York, New York; Columbia University, New York, New York; NYU Grossman School of Medicine, New York, New York; NYC Health+Hospitals, Brooklyn, NY

## Abstract

**Background:**

There have been over 34,000 mpox cases identified in the United States during the recent mpox outbreak, with New York City (NYC) being the epicenter since 2022. At the outset, limited information and resources were available for health care providers (HCPs) to treat individuals experiencing severe mpox manifestations. Due to the NYC outbreak primarily impacting sexual minority men (SMM), compounding concerns about inequitable health care treatment experiences, this qualitative study aims to understand the experiences of diverse SMM patients with severe mpox when treated by HCPs in NYC during the mpox outbreak.

**Table 1:**
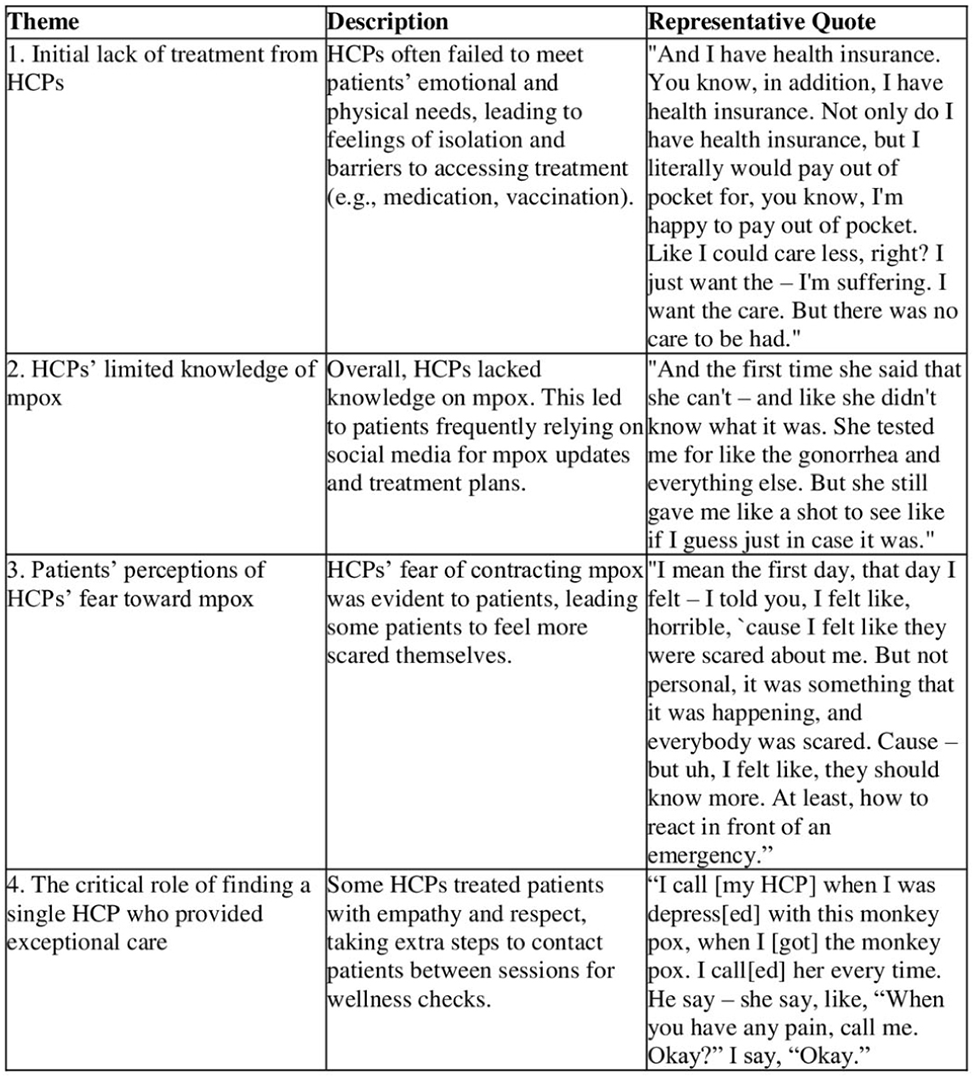
Overview of key themes, their descriptions, and representative quotes.

**Methods:**

Between March and December 2024, participants living in NYC who had a past diagnosis of mpox (N = 20; 26-60y, Mage = 39.4±10y; 80% identifying as homosexual; 45% Black, 35% White; 45% Hispanic/Latino) completed semi-structured interviews. Interviews took place in-person or virtually and were conducted in English and Spanish. Two research assistants completed analyses using template-style thematic analysis. The current analysis used a subset of the data specific to HCP interactions.

**Results:**

Four major themes were identified: 1) Initial lack of treatment from HCPs, 2) HCPs’ limited knowledge of mpox, 3) patients’ perceptions of HCPs’ fear toward mpox, and 4) the critical role of finding a single HCP who provided exceptional care. Descriptions and representative quotes for each of these themes are displayed in Table 1.

**Conclusion:**

Overall, SMM participants felt the health care system, specifically at the level of patient-provider interactions, failed them during the mpox outbreak. Yet, even amidst a public health crisis in which there was limited knowledge on best treatment practices, participants identified alternative strategies to make up for what was lacking in their initial interactions with HCPs. HCPs with whom participants reported positive experiences consistently displayed frequent, effective, and empathetic communication and proactively checked in to support continuity of care. Future research would benefit from exploring how patients’ intersectional identities (i.e., SMM of minoritized racial and ethnic backgrounds) may have impacted their experiences with HCPs.

**Disclosures:**

Ofole Mgbako, MD, MS, Gilead Sciences: Advisor/Consultant

